# Sequential Hybrid CryoMaze Ablation versus Surgical CryoMaze Alone for the Treatment of Atrial Fibrillation (SurHyb): study protocol for a randomized controlled trial

**DOI:** 10.1186/s13063-016-1634-4

**Published:** 2016-10-24

**Authors:** Martin Eisenberger, Alan Bulava, Josef Kautzner, Petr Neuzil, Ales Mokracek, Jiri Hanis, Ladislav Dusek

**Affiliations:** 1 South Bohemia Cardiac Centre, Budweis Hospital, B. Nemcove 54, Budweis, Czech Republic; 2 Faculty of Health and Social Studies, South Bohemia University, J. Boreckeho 27, Budweis, Czech Republic; 3 Department of Cardiology, Institute for Clinical and Experimental Medicine, Videnska 1958/9, Prague, Czech Republic; 4 Department of Cardiology, Na Homolce Hospital, Roentgenova 2, Prague, Czech Republic; 5 The Institute of Biostatistics and Analyses, Masaryk University, Kamenice 5, Brno, Czech Republic; 6 Faculty of Medicine and Dentistry, Palacky University Olomouc, Olomouc, Czech Republic

**Keywords:** Atrial fibrillation, Catheter ablation, Surgical ablation, CryoMaze, Randomized controlled clinical trial, Hybrid approach

## Abstract

**Background:**

Atrial fibrillation is common in patients with structural heart disease who are undergoing cardiac surgery. Surgical CryoMaze has been shown to be an effective treatment in several trials, but success rates have varied considerably, between 47–95 %. The sequential hybrid approach, combining surgical CryoMaze followed by radiofrequency catheter ablation, can achieve high freedom from atrial arrhythmias, even when rigorous methods to detect arrhythmias after the procedure are used. However, data from randomized trials comparing hybrid ablations to surgical ablations alone are lacking.

**Methods/Design:**

The SurHyb study is a prospective, multicenter, randomized study. Patients with persistent or long-standing persistent atrial fibrillation will be randomized to either surgical CryoMaze alone or surgical CryoMaze followed by catheter ablation 3 months post-surgery. The primary outcome measure is arrhythmia-free survival without class I or III antiarrhythmic drugs, which will be evaluated using 7-day ECG Holter monitoring at 24 months. A total of 260 patients will be investigated from three medical centers in the Czech Republic to obtain the relevant information.

**Discussion:**

This is the first randomized study that compares surgical CryoMaze alone with the staged hybrid surgical CryoMaze followed by catheter ablation in patients with persistent or long-standing persistent atrial fibrillation. These results will contribute to the optimization of the treatment for these patients.

**Trial registration:**

Czech Clinical Trials Registry, cz-301020151253. Registered on 30 October 2015.

## Background

Atrial fibrillation (AF), the most common clinically significant cardiac arrhythmia, is associated with increased mortality and morbidity [1]. The incidence of AF will continue to increase due to aging of the population. In patients indicated for cardiac surgery, the prevalence of AF is higher compared to the general population [2], and it can be as high as 50 % in individuals with mitral valve disease [3].

Both surgical and catheter ablation have become recognized treatments for symptomatic and drug-refractory AF. The CryoMaze ablation procedure was developed as a surgical alternative to cut-and-sew procedures as a way to decrease invasiveness [4]. A variety of surgical ablation energy sources have been used including microwaves, laser, and unipolar or bipolar radiofrequency energy. The CryoMaze operation is usually done in conjunction with a coronary bypass operation, valve surgery, or other open heart surgery. Incomplete lines after surgical ablation are common and can be pro-arrhythmic [5, 6]. The efficacy of the CryoMaze procedure as the sole treatment for persistent AF has been shown in several studies, but the results are hindered by small patient numbers and less than accurate follow-up assessments of heart rhythm, such as via telephone interviews with only occasional use of continuous electrocardiographic (ECG) recordings [4, 7]. This has led to mixed results, with rates of freedom from AF being reported between 47–95 % [8, 9]. Additionally, the need for subsequent transvenous catheter procedures remains unknown; also, the clinical procedures employed varied between centers.

In a non-randomized study, sequential surgical CryoMaze procedures followed by catheter ablation led to high freedom from arrhythmia in patients with persistent and long-standing persistent AF and without an increase in complications [10]. However, there have been no randomized clinical trials conducted to evaluate whether the hybrid approach is superior to a CryoMaze procedure alone; therefore, a recommendation regarding the best procedure for all patients is unavailable.

The aim of this randomized, multicenter clinical trial is to compare clinical efficacy and safety of the staged hybrid ablation to a CryoMaze procedure alone in a well-described population of patients with AF.

## Methods

**Fig. 1:**
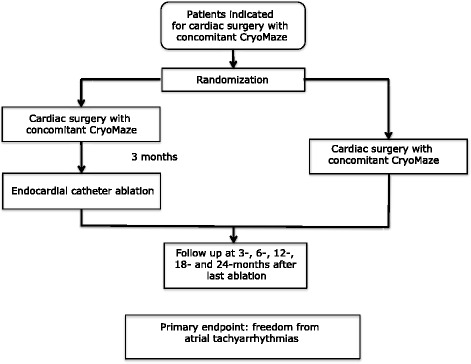
SurHyb study flowchart

The Sequential Hybrid Surgical CryoMaze Ablation versus Surgical CryoMaze Alone for the Treatment of Atrial Fibrillation (SurHyb) study was designed to be a prospective, randomized multicenter study. Patients will be recruited from three major cardiovascular centers in the Czech Republic (South Bohemia Cardiac Centre in Budweis, the Institute for Clinical and Experimental Medicine in Prague, and Na Homolce Hospital in Prague). Patients will be approached during the clinical review before the surgical operation and will be offered an opportunity to participate in the study. There will be a follow-up period of 2 years for each enrolled patient. The objective of the study is to compare a staged hybrid surgical cryoablation, followed 3 months later by catheter ablation, to a surgical CryoMaze alone. A flowchart of the SurHyb trial is shown in Fig. 1. Written informed consent will be obtained from all patients prior to the study. All participating centers will have an appointed local investigator who will be responsible for appropriate inclusion and follow-up of patients. The trial sponsor is the South Bohemia University in Budweis. The regulations regarding medical confidentiality and data protection are fulfilled.

### Inclusion and exclusion criteria

All patients need to meet the following inclusion criteria:

1. Cardiac surgery (either valve operation or coronary artery bypass grafting or combination of both) with concomitant CryoMaze ablation as a treatment for persistent or long-standing persistent AF
2. At least one failed class I or III antiarrhythmic drug
3. Age ≥18 years
4. Willing to sign the informed consent form
5. Symptomatic persistent or long-standing persistent AF documented using electrocardiography; symptoms may include, but are not restricted to, palpitations, shortness of breath, chest pain, fatigue, left ventricular dysfunction, or any combination of these
6. Able and willing to comply with all pre-, post-, and follow-up testing and requirements

The exclusion criteria are as follows:

1. Permanent and paroxysmal AF
2. Previous surgical or catheter ablation for AF (does not include ablation for atrial flutter or other supraventricular arrhythmia)
3. Clinically estimated life expectancy of 5 years or less or mental and/or physical inability to comply with the follow-up period
4. Arrhythmia secondary to a reversible or non-cardiac cause
5. Serious liver or renal dysfunction (alanine aminotransferase [ALT] >80 U/l, aspartate aminotransferase [AST] >80 U/l, creatinine >132 μmol/l)
6. Pregnancy or the possibility of pregnancy, or breast feeding
7. Age younger than 18 years
8. Contraindication to systemic anticoagulation
9. Enrollment in an experimental study evaluating another device or drug under investigation
10. Patient not competent to legally represent him or herself (e.g., requires a guardian or caretaker as a legal representative)

### Screening and enrollment

Approximately 600 patients per year are scheduled for cardiac surgery with concomitant CryoMaze ablation in all three cardiovascular centers involved in the study. The patients who fulfill the inclusion criteria will be approached by the local investigator, who will explain the SurHyb study and provide a patient information form. We expect that 50–70 % of eligible patients will consent.

### Randomization

The SurHyb study is a prospective, randomized, multicenter study. After providing informed consent, patients will be randomly assigned using a computer system to one of the two groups — the hybrid group or the surgery alone group — initially in a 1:1 ratio. The surgeon will be blinded as to the treatment group until the surgery is completed. All patient data will be collected using a standard form and transmitted to a central database.

### Interventions

The study consists of two study arms: the *hybrid group*, which will receive surgical CryoMaze followed, in 3 months, by catheter ablation in all patients, or the *surgical CryoMaze group*, in which participants will receive surgical cryoablation with no planned catheter ablation after discharge.

### Primary outcome measure

The primary outcome measure is arrhythmia-free survival without class I or III antiarrhythmic drugs, which will be evaluated using a 7-day Holter monitoring at 24 months. After a 3-month blanking period, if a patient requires a repeat ablation, cardioversion, or antiarrhythmic drugs, this will be considered as a failure.

### Secondary outcome measures

The secondary outcome measures involve arrhythmia-free survival without class I or III antiarrhythmic drugs and without a need for repeat ablation or cardioversion, evaluated using a 7-day Holter monitoring at 6, 12, and 18 months. The measures include:

- Arrhythmia-free survival regardless of antiarrhythmic drugs status or re-do ablations
- Number of cardioversions
- Number of re-do ablations
- Frequency and duration of hospital admissions for recurrent arrhythmias
- Frequency and description of any procedure-related complications (during both surgical and catheter ablation)
- Cardiovascular mortality and morbidity
- Changes of left atrial dimension as measured using ultrasound echocardiography

### Anticoagulation therapy before ablation

Patients on warfarin, apixaban, dabigatran, or rivaroxaban will stop the medication and switch to either unfractionated or low molecular weight heparin, which will be maintained during the surgical procedure. Novel oral anticoagulants can only be used in accordance with current recommendations during the trial [11]. If the patient is taking only aspirin, it may or may not need to be discontinued.

### Surgical ablation

The CryoMaze ablation will be performed as an adjunct to a coronary artery bypass, valve surgery, or a combination of both. It will be done using the standard sternotomy approach, and each lesion will be created by an application of cryothermia at a fixed temperature. The target temperature, duration of application, and location of each lesion will be recorded. The standard minimum CryoMaze protocol will consist of continuous lesions surrounding each group of right and left pulmonary veins with linear lesions between the superior and inferior pulmonary veins with the aim of creating a box lesion. Other lines can be created at the surgeon’s discretion. The left atrial appendage will be excluded in patients with a previous history of stroke or a CHA_2_DS_2_-VASc score ≥2. All patients who remain in AF after the surgical procedure is completed will undergo attempted cardioversion before chest closure and again before hospital discharge if the arrhythmia persists. Patients will be left on their current antiarrhythmic medication covering the 3-month blanking period to avoid early recurrences, with the aim of discontinuing the medication at the first follow-up visit. Recurrences of AF, atrial tachycardias, or atrial flutter during the first 3 months may require a change of the antiarrhythmic medication or direct current cardioversion, but this will not be counted against the primary or secondary endpoints. The patients will continue with the same anticoagulation therapy after the operation unless a change in the medication has been required during the admission (e.g., anticoagulation for a newly implanted artificial valve or discontinuation of anticoagulation for severe bleeding). A 7-day ECG Holter monitor will be scheduled 3 months after surgery (just before admission for the catheter ablation in the hybrid group). If a patient in the surgical cryoablation group is in persistent AF after the 3-month blanking period, the patient will receive direct current cardioversion, and if he or she remains in normal sinus rhythm, this will not be considered a failure.

### Catheter ablation

Patients randomized to the hybrid treatment arm will be admitted for a staged catheter ablation treatment 3 months after the CryoMaze procedure. In general, patients will continue with warfarin or stop one of the novel anticoagulation drugs 24–48 hours before the planned procedure (unless the surgeon decides otherwise). Electro-anatomical mapping of the left and right atria will be performed using a CARTO navigation system. First, an anatomical map will be created to provide a first look at the actual locations of the CryoMaze ablation lines (regions with voltage <0.1 mV). Pulmonary vein isolation will be confirmed by the absence of signals recorded using a multielectrode circular catheter. Completeness of all linear lesions created during the surgical CryoMaze will be confirmed by a bidirectional conduction block across the relevant line. Any gaps will be re-ablated using radiofrequency catheter ablation. If the patient is in atrial tachycardia at the beginning of the procedure, the tachycardia will be mapped and ablated as the first step. If the patient is in AF, an external electrical cardioversion will be performed during the procedure to restore sinus rhythm. The investigator can then add any of the current ablation methods including substrate modification or ablation of triggers induced by an isoproterenol infusion. Inducibility of AF or atrial tachycardia at the end of the procedure will be recorded. Patients will be discharged on their current antiarrhythmic medication for the 3-month blanking period with the aim of discontinuing the medication at the first follow-up visit. Any atrial tachyarrhythmias that may occur during the blanking period after the catheter ablation may require a change of the antiarrhythmic medication or external cardioversion. Intravenous heparin will be administered during the procedure, and warfarin will continue post-operatively with a target international normalized ratio between 2.0 and 3.0. Alternatively, novel oral anticoagulants, such as dabigatran, apixaban, or rivaroxaban, will be prescribed for a period of at least 3 months.

### Follow-up

Clinical follow-up will be performed at 3, 6, 12, 18, and 24 months after the index procedure (surgical ablation in the surgical cryoablation group and catheter ablation in the hybrid group). Seven-day Holter monitoring will be performed before each follow-up in all patients. The absence or presence of atrial tachyarrhythmias on the ECG Holter will be assessed, and any need for additional ablation procedures, antiarrhythmic drugs, or cardioversions after the blanking period will be considered as a failure. Clinical status, medication, blood pressure, 12-lead electrocardiogram, and any adverse events will be documented during every visit. Unless necessary, antiarrhythmic drug therapy will be discontinued at the first follow-up visit. Medical records will be reviewed at each visit to determine the moment of antiarrhythmic medication changes.

An echocardiogram will be performed within 1 week following the surgical ablation or, if performed, within 1 week after the catheter ablation. Further echocardiograms will be performed at the 12 and 24 month follow-ups. Left atrial size will be determined using the long-axis parasternal view and the apical two-chamber view. In addition, the left ventricular end systolic and the left ventricular end diastolic diameter will be measured, and diastolic function will be assessed by computing the ratio of early to late diastolic filling, the E/A ratio.

### Withdrawal of trial participants

Participants can withdraw from the trial at any time for any reason without their medical care being affected. Data already collected will continue to be used, and the patients will be asked if they are still willing to provide follow-up data. The reason for withdrawal will be documented whenever possible.

### Statistical methods

Absolute values of continuous variables will be expressed using standard summary statistics, arithmetic mean, standard deviation, and corresponding 95 % confidence interval. The study is designed to compare recurrence rates of AF or atrial tachycardia episodes at 24 months between two treatment groups as the primary endpoint. The arrhythmia-free survival rate is defined as the percentage of patients free of atrial arrhythmias. Fisher’s exact test will be used for this categorical data to assess the difference in frequency of arrhythmia recurrence. The odds ratio and its 95 % confidence interval will be estimated as a quantitative measure of risk in the association frequency table. Differences in continuous data describing duration of arrhythmia recurrences will be tested using Student’s *t* test. Kaplan-Meier curves will be plotted to compare event-free survival time between both groups. The log-rank test will be performed to compare arrhythmia-free survival time distributions between the study arms. Influence of mutually independent risk factors will be quantified on the basis of the hazard ratio (HR) and its 95 % confidence interval. Both univariate and multivariate adjusted HRs will be estimated using the Cox proportional hazard models with an adopted forward stepwise algorithm. All variables will be analyzed on the basis of the intention-to-treat protocol. A *P* value less than 0.05 will be considered as a boundary of statistical significance of differences in all performed tests. Analyses will be performed using SPSS version 16.0 (SPSS Inc., Chicago, IL, USA).

### Sample size calculation

Power calculations to provide estimates for the necessary sample size were conducted concerning recurrence rates of AF or atrial tachycardia episodes at 24 months as the primary outcome endpoint. The clinical efficacy of the hybrid ablation procedure was assumed to be 86 %, whereas the efficacy of the surgical ablation alone was assumed to be 50–70 %. To ensure 80 % power of the Fisher exact test at the 5 % significance level, a sample size of at least 130 must be included in each of the trial arms. Considering a dropout rate of 20 %, approximately 170 patients will finally be recruited for each trial arm. At the end of the study, the number of patients free from arrhythmias should be similar in both groups, but the proportion of those submitted to another procedure, commenced on antiarrhythmic drugs, or undergoing cardioversion should differ significantly.

### Data management

A tailor-made website has been developed for the SurHyb study. Each participating medical center will have access to a dedicated part of the website. After enrollment and randomization of participants, data from medical records, drug regimens, interviews, and electrocardiograms will be added to the website. The website will be updated after each outpatient clinical visit during the follow-up period. The follow-up status of all patients can be viewed instantly.

At the end of the study, all data will be entered and stored on a password-protected computer. A data monitoring committee, of which at least two members will be independent of the research team, will monitor the data management process regularly. All data will be frozen and locked to prevent further editing after validation by the data monitoring committee. Only the data monitoring committee, the study research assistant, and the principal investigator will have access to the final data set. This committee will meet twice per year. Using statistical criteria for acceptable deviations from the null hypothesis, the committee will advise whether the recruitment can continue or whether the study should be terminated. The final analysis will be performed after all the patients complete their 2-year follow-up.

Reasons for dropouts and the number of patients in each group will be reported. This information will be used to see how missing data will be handled in statistical analysis. Data already collected will continue to be used and, if possible, post-dropout data will be collected on the main endpoints. In case of a high percentage of missing data, imputation techniques might be required.

### Safety monitoring

The local investigator at each site will review safety data for the duration of the trial. Serious adverse events are defined as life-threatening events or events resulting in death or hospitalization. All serious adverse events linked with the study will be reported to the Budweis Hospital research ethics committee within 24 hours of study staff becoming aware of the event and in accordance with the institutional research ethics committee requirements. Serious adverse events will be completed with detailed information, such as event description, date of onset and resolution, severity, and action taken.

## Discussion

This randomized controlled trial is the first of its kind in which hybrid ablation will be compared with surgical CryoMaze alone to compare differences relative to freedom from atrial tachyarrhythmia.

CryoMaze ablation is a less invasive modification of the original cut-and-sew method using a cryoprobe to create ablation lesions. Success rates have been reported to be as high as 95 % at 12 months; however, follow-ups were conducted through telephone interviews only [4]. When more accurate methods to determine heart rhythm were used, such as ECG Holter monitors over several days, success rates fell to 76 % at 12 months [9]. Other authors have reported less promising outcomes. In one study, only 47 % of patients with persistent AF who underwent surgical CryoMaze with concomitant cardiac surgery were in long-term normal sinus rhythm [8].

Hybrid ablations may overcome the limitations associated with both surgical and transvenous endocardial ablations alone. Neither of the approaches alone can guarantee complete transmurality, which is crucial, particularly with regard to persistent AF requiring relatively long continuous lesions. It is also possible to ablate arrhythmia triggers and inducible atrial tachycardias after the surgical ablation; these would otherwise be left untreated. Studies published to date included simultaneous or sequential hybrid procedures with reported success rates from 83–94 %, but the experience has only been with bipolar or monopolar radiofrequency energy [12–15]. The only report on hybrid ablations with cryoenergy used during the surgical part of the procedure revealed an overall freedom from atrial tachyarrhythmias to be 86 % at 12 months. The overall success rate at 1 year can reach 94 % if patients on antiarrhythmic drugs or patients who underwent a successful re-do ablation are not excluded as a failure [10]. There is certainly reporting bias among published studies, as several large studies of hybrid AF ablation, including the staged Dual Epicardial Endocardial Persistent (DEEP) AF study, FAST-II, and SCALA have not yet been published.

Data from randomized trials comparing hybrid ablations to either surgical or catheter ablation are lacking, but several randomized clinical trials are ongoing. The staged transthoracic approach to persistent AF (TOP-AF) study is a randomized controlled trial comparing percutaneous catheter ablation with surgical ablation as the first procedure followed by a second procedure, if necessary [16]. The researchers anticipate that the use of a staged strategy combining surgical and percutaneous approaches might be more favorable in treatment of persistent AF than a single percutaneous ablation. Another ongoing trial (Prospective, Randomized Comparison of Hybrid Ablation vs. Catheter Ablation, PRHACA) will compare endocardial catheter ablation using the Arctic Front CryoAblation catheter (Medtronic, Minneapolis, MN, USA) to isolate the pulmonary veins and a Navistar Smart Touch catheter (Biosense Webster, Diamond Bar, CA, USA) to create ablation lines during the hybrid surgical ablation using a VisiTrax ablation device (nContact, Morrisville, NC, USA) for the surgical portion of the procedure. As far as we know, there are no ongoing randomized trials using cryoenergy for the surgical part of the hybrid procedure.

## Trial status

The trial is currently in the recruitment phase.

## Abbreviations

AF: Atrial fibrillation
E/A ratio: Early to late diastolic filling ratio
ECG: Electrocardiographic
PRHACA Prospective: Randomized Comparison of Hybrid Ablation vs. Catheter Ablation
